# Supplementary material for: Pyruvate Dehydrogenase Kinase 1 inhibition mediated oxidative phosphorylation enhancement in cartilage promotes osteoarthritis progression
Source: BMC Musculoskelet Disord. 2023 Jul 20;24:597. doi: 10.1186/s12891-023-06585-6 (PMC10357736; doi:10.1186/s12891-023-06585-6)
Supplement: Supplementary file 1 — Additional file 1. [file 12891_2023_6585_MOESM1_ESM.zip › KEGG.Gsea.1653622432464/KEGG_NEUROTROPHIN_SIGNALING_PATHWAY.html]

Details for gene set KEGG\_NEUROTROPHIN\_SIGNALING\_PATHWAY[GSEA]

|  || Dataset | 55457\_collapsed\_to\_symbols.cls.cls #OA\_versus\_normal.cls.cls #OA\_versus\_normal\_repos |
| Phenotype | cls.cls#OA\_versus\_normal\_repos |
| Upregulated in class | normal |
| GeneSet | KEGG\_NEUROTROPHIN\_SIGNALING\_PATHWAY |
| Enrichment Score (ES) | -0.29850057 |
| Normalized Enrichment Score (NES) | -1.323985 |
| Nominal p-value | 0.052005943 |
| FDR q-value | 0.26289114 |
| FWER p-Value | 0.999 |
Table: GSEA Results Summary

  

Fig 1: Enrichment plot: KEGG\_NEUROTROPHIN\_SIGNALING\_PATHWAY      
 Profile of the Running ES Score & Positions of GeneSet Members on the Rank Ordered List

  

| SYMBOL | TITLE | RANK IN GENE LIST | RANK METRIC SCORE | RUNNING ES | CORE ENRICHMENT || 1 | YWHAH | tyrosine 3-monooxygenase/tryptophan 5-monooxygenase activation protein eta [Source:HGNC Symbol;Acc:HGNC:12853] | 118 | 0.889 | 0.0134 | No |
| 2 | PSEN1 | presenilin 1 [Source:HGNC Symbol;Acc:HGNC:9508] | 425 | 0.695 | 0.0072 | No |
| 3 | BEX3 | brain expressed X-linked 3 [Source:HGNC Symbol;Acc:HGNC:13388] | 438 | 0.690 | 0.0238 | No |
| 4 | NFKBIE | NFKB inhibitor epsilon [Source:HGNC Symbol;Acc:HGNC:7799] | 626 | 0.613 | 0.0248 | No |
| 5 | MAPK9 | mitogen-activated protein kinase 9 [Source:HGNC Symbol;Acc:HGNC:6886] | 721 | 0.588 | 0.0324 | No |
| 6 | PLCG2 | phospholipase C gamma 2 [Source:HGNC Symbol;Acc:HGNC:9066] | 725 | 0.588 | 0.0472 | No |
| 7 | MAPK3 | mitogen-activated protein kinase 3 [Source:HGNC Symbol;Acc:HGNC:6877] | 757 | 0.579 | 0.0595 | No |
| 8 | CAMK2A | calcium/calmodulin dependent protein kinase II alpha [Source:HGNC Symbol;Acc:HGNC:1460] | 916 | 0.538 | 0.0608 | No |
| 9 | CALM3 | calmodulin 3 [Source:HGNC Symbol;Acc:HGNC:1449] | 1039 | 0.510 | 0.0643 | No |
| 10 | MAGED1 | MAGE family member D1 [Source:HGNC Symbol;Acc:HGNC:6813] | 1128 | 0.491 | 0.0699 | No |
| 11 | AKT1 | AKT serine/threonine kinase 1 [Source:HGNC Symbol;Acc:HGNC:391] | 1192 | 0.478 | 0.0771 | No |
| 12 | CALML5 | calmodulin like 5 [Source:HGNC Symbol;Acc:HGNC:18180] | 1261 | 0.465 | 0.0836 | No |
| 13 | MAP2K5 | mitogen-activated protein kinase kinase 5 [Source:HGNC Symbol;Acc:HGNC:6845] | 1391 | 0.444 | 0.0849 | No |
| 14 | NTF3 | neurotrophin 3 [Source:HGNC Symbol;Acc:HGNC:8023] | 1398 | 0.443 | 0.0957 | No |
| 15 | CRK | "CRK proto-oncogene, adaptor protein [Source:HGNC Symbol;Acc:HGNC:2362]" | 2018 | 0.360 | 0.0564 | No |
| 16 | PIK3CB | "phosphatidylinositol-4,5-bisphosphate 3-kinase catalytic subunit beta [Source:HGNC Symbol;Acc:HGNC:8976]" | 2186 | 0.340 | 0.0520 | No |
| 17 | PRKCD | protein kinase C delta [Source:HGNC Symbol;Acc:HGNC:9399] | 2237 | 0.335 | 0.0566 | No |
| 18 | CRKL | "CRK like proto-oncogene, adaptor protein [Source:HGNC Symbol;Acc:HGNC:2363]" | 2283 | 0.329 | 0.0615 | No |
| 19 | MAPK14 | mitogen-activated protein kinase 14 [Source:HGNC Symbol;Acc:HGNC:6876] | 2352 | 0.320 | 0.0643 | No |
| 20 | SHC1 | SHC adaptor protein 1 [Source:HGNC Symbol;Acc:HGNC:10840] | 2485 | 0.304 | 0.0617 | No |
| 21 | MAPK13 | mitogen-activated protein kinase 13 [Source:HGNC Symbol;Acc:HGNC:6875] | 2544 | 0.298 | 0.0648 | No |
| 22 | CAMK2G | calcium/calmodulin dependent protein kinase II gamma [Source:HGNC Symbol;Acc:HGNC:1463] | 2900 | 0.261 | 0.0436 | No |
| 23 | NTRK1 | neurotrophic receptor tyrosine kinase 1 [Source:HGNC Symbol;Acc:HGNC:8031] | 3015 | 0.248 | 0.0410 | No |
| 24 | FRS2 | fibroblast growth factor receptor substrate 2 [Source:HGNC Symbol;Acc:HGNC:16971] | 3385 | 0.213 | 0.0176 | No |
| 25 | RPS6KA1 | ribosomal protein S6 kinase A1 [Source:HGNC Symbol;Acc:HGNC:10430] | 3389 | 0.213 | 0.0228 | No |
| 26 | SORT1 | sortilin 1 [Source:HGNC Symbol;Acc:HGNC:11186] | 3481 | 0.204 | 0.0208 | No |
| 27 | CAMK4 | calcium/calmodulin dependent protein kinase IV [Source:HGNC Symbol;Acc:HGNC:1464] | 3664 | 0.189 | 0.0114 | No |
| 28 | RPS6KA4 | ribosomal protein S6 kinase A4 [Source:HGNC Symbol;Acc:HGNC:10433] | 3702 | 0.185 | 0.0132 | No |
| 29 | BAD | BCL2 associated agonist of cell death [Source:HGNC Symbol;Acc:HGNC:936] | 3788 | 0.178 | 0.0111 | No |
| 30 | FASLG | Fas ligand [Source:HGNC Symbol;Acc:HGNC:11936] | 3931 | 0.162 | 0.0041 | No |
| 31 | RELA | "RELA proto-oncogene, NF-kB subunit [Source:HGNC Symbol;Acc:HGNC:9955]" | 3942 | 0.161 | 0.0074 | No |
| 32 | ARHGDIB | Rho GDP dissociation inhibitor beta [Source:HGNC Symbol;Acc:HGNC:679] | 4085 | 0.147 | 0.0001 | No |
| 33 | NGFR | nerve growth factor receptor [Source:HGNC Symbol;Acc:HGNC:7809] | 4156 | 0.142 | -0.0018 | No |
| 34 | MAPK7 | mitogen-activated protein kinase 7 [Source:HGNC Symbol;Acc:HGNC:6880] | 4396 | 0.121 | -0.0174 | No |
| 35 | NFKB1 | nuclear factor kappa B subunit 1 [Source:HGNC Symbol;Acc:HGNC:7794] | 4626 | 0.102 | -0.0327 | No |
| 36 | RIPK2 | receptor interacting serine/threonine kinase 2 [Source:HGNC Symbol;Acc:HGNC:10020] | 4848 | 0.083 | -0.0479 | No |
| 37 | SH2B3 | SH2B adaptor protein 3 [Source:HGNC Symbol;Acc:HGNC:29605] | 4960 | 0.076 | -0.0546 | No |
| 38 | ZNF274 | zinc finger protein 274 [Source:HGNC Symbol;Acc:HGNC:13068] | 5181 | 0.058 | -0.0704 | No |
| 39 | TP73 | tumor protein p73 [Source:HGNC Symbol;Acc:HGNC:12003] | 5222 | 0.055 | -0.0721 | No |
| 40 | NTRK2 | neurotrophic receptor tyrosine kinase 2 [Source:HGNC Symbol;Acc:HGNC:8032] | 5431 | 0.038 | -0.0874 | No |
| 41 | TP53 | tumor protein p53 [Source:HGNC Symbol;Acc:HGNC:11998] | 5511 | 0.032 | -0.0928 | No |
| 42 | BCL2 | BCL2 apoptosis regulator [Source:HGNC Symbol;Acc:HGNC:990] | 5609 | 0.023 | -0.0998 | No |
| 43 | GRB2 | growth factor receptor bound protein 2 [Source:HGNC Symbol;Acc:HGNC:4566] | 5697 | 0.016 | -0.1062 | No |
| 44 | SHC2 | SHC adaptor protein 2 [Source:HGNC Symbol;Acc:HGNC:29869] | 5738 | 0.012 | -0.1090 | No |
| 45 | TRAF6 | TNF receptor associated factor 6 [Source:HGNC Symbol;Acc:HGNC:12036] | 5761 | 0.011 | -0.1104 | No |
| 46 | YWHAZ | tyrosine 3-monooxygenase/tryptophan 5-monooxygenase activation protein zeta [Source:HGNC Symbol;Acc:HGNC:12855] | 5770 | 0.010 | -0.1108 | No |
| 47 | IKBKB | inhibitor of nuclear factor kappa B kinase subunit beta [Source:HGNC Symbol;Acc:HGNC:5960] | 5914 | 0.001 | -0.1220 | No |
| 48 | KIDINS220 | kinase D interacting substrate 220 [Source:HGNC Symbol;Acc:HGNC:29508] | 6101 | -0.013 | -0.1362 | No |
| 49 | IRS4 | insulin receptor substrate 4 [Source:HGNC Symbol;Acc:HGNC:6128] | 6106 | -0.013 | -0.1362 | No |
| 50 | RAF1 | "Raf-1 proto-oncogene, serine/threonine kinase [Source:HGNC Symbol;Acc:HGNC:9829]" | 6187 | -0.019 | -0.1419 | No |
| 51 | CALML3 | calmodulin like 3 [Source:HGNC Symbol;Acc:HGNC:1452] | 6348 | -0.032 | -0.1537 | No |
| 52 | RAP1A | "RAP1A, member of RAS oncogene family [Source:HGNC Symbol;Acc:HGNC:9855]" | 6580 | -0.048 | -0.1705 | No |
| 53 | ARHGDIA | Rho GDP dissociation inhibitor alpha [Source:HGNC Symbol;Acc:HGNC:678] | 6664 | -0.053 | -0.1756 | No |
| 54 | MAPK1 | mitogen-activated protein kinase 1 [Source:HGNC Symbol;Acc:HGNC:6871] | 6853 | -0.068 | -0.1886 | No |
| 55 | RAP1B | "RAP1B, member of RAS oncogene family [Source:HGNC Symbol;Acc:HGNC:9857]" | 6980 | -0.078 | -0.1965 | No |
| 56 | MAP3K1 | mitogen-activated protein kinase kinase kinase 1 [Source:HGNC Symbol;Acc:HGNC:6848] | 7066 | -0.085 | -0.2010 | No |
| 57 | RPS6KA3 | ribosomal protein S6 kinase A3 [Source:HGNC Symbol;Acc:HGNC:10432] | 7092 | -0.087 | -0.2007 | No |
| 58 | RAC1 | Rac family small GTPase 1 [Source:HGNC Symbol;Acc:HGNC:9801] | 7192 | -0.096 | -0.2060 | No |
| 59 | NRAS | "NRAS proto-oncogene, GTPase [Source:HGNC Symbol;Acc:HGNC:7989]" | 7300 | -0.102 | -0.2118 | No |
| 60 | PIK3CD | "phosphatidylinositol-4,5-bisphosphate 3-kinase catalytic subunit delta [Source:HGNC Symbol;Acc:HGNC:8977]" | 7405 | -0.111 | -0.2171 | No |
| 61 | NGF | nerve growth factor [Source:HGNC Symbol;Acc:HGNC:7808] | 7572 | -0.125 | -0.2269 | No |
| 62 | BDNF | brain derived neurotrophic factor [Source:HGNC Symbol;Acc:HGNC:1033] | 7705 | -0.134 | -0.2338 | No |
| 63 | PIK3CA | "phosphatidylinositol-4,5-bisphosphate 3-kinase catalytic subunit alpha [Source:HGNC Symbol;Acc:HGNC:8975]" | 7733 | -0.136 | -0.2325 | No |
| 64 | PIK3CG | "phosphatidylinositol-4,5-bisphosphate 3-kinase catalytic subunit gamma [Source:HGNC Symbol;Acc:HGNC:8978]" | 7953 | -0.152 | -0.2457 | No |
| 65 | HRAS | "HRas proto-oncogene, GTPase [Source:HGNC Symbol;Acc:HGNC:5173]" | 7977 | -0.153 | -0.2436 | No |
| 66 | AKT3 | AKT serine/threonine kinase 3 [Source:HGNC Symbol;Acc:HGNC:393] | 8008 | -0.156 | -0.2420 | No |
| 67 | SOS2 | SOS Ras/Rho guanine nucleotide exchange factor 2 [Source:HGNC Symbol;Acc:HGNC:11188] | 8058 | -0.160 | -0.2418 | No |
| 68 | RPS6KA6 | ribosomal protein S6 kinase A6 [Source:HGNC Symbol;Acc:HGNC:10435] | 8071 | -0.161 | -0.2386 | No |
| 69 | MAPK12 | mitogen-activated protein kinase 12 [Source:HGNC Symbol;Acc:HGNC:6874] | 8161 | -0.168 | -0.2413 | No |
| 70 | BRAF | "B-Raf proto-oncogene, serine/threonine kinase [Source:HGNC Symbol;Acc:HGNC:1097]" | 8171 | -0.169 | -0.2377 | No |
| 71 | RPS6KA2 | ribosomal protein S6 kinase A2 [Source:HGNC Symbol;Acc:HGNC:10431] | 8397 | -0.185 | -0.2506 | No |
| 72 | RHOA | ras homolog family member A [Source:HGNC Symbol;Acc:HGNC:667] | 8601 | -0.201 | -0.2614 | No |
| 73 | YWHAQ | tyrosine 3-monooxygenase/tryptophan 5-monooxygenase activation protein theta [Source:HGNC Symbol;Acc:HGNC:12854] | 8675 | -0.207 | -0.2618 | No |
| 74 | CAMK2B | calcium/calmodulin dependent protein kinase II beta [Source:HGNC Symbol;Acc:HGNC:1461] | 8782 | -0.215 | -0.2646 | No |
| 75 | GAB1 | GRB2 associated binding protein 1 [Source:HGNC Symbol;Acc:HGNC:4066] | 8786 | -0.215 | -0.2594 | No |
| 76 | PIK3R3 | phosphoinositide-3-kinase regulatory subunit 3 [Source:HGNC Symbol;Acc:HGNC:8981] | 8801 | -0.217 | -0.2549 | No |
| 77 | PIK3R5 | phosphoinositide-3-kinase regulatory subunit 5 [Source:HGNC Symbol;Acc:HGNC:30035] | 8804 | -0.217 | -0.2496 | No |
| 78 | MAPK8 | mitogen-activated protein kinase 8 [Source:HGNC Symbol;Acc:HGNC:6881] | 8956 | -0.228 | -0.2556 | No |
| 79 | CSK | C-terminal Src kinase [Source:HGNC Symbol;Acc:HGNC:2444] | 9056 | -0.236 | -0.2573 | No |
| 80 | ABL1 | "ABL proto-oncogene 1, non-receptor tyrosine kinase [Source:HGNC Symbol;Acc:HGNC:76]" | 9081 | -0.238 | -0.2531 | No |
| 81 | BAX | "BCL2 associated X, apoptosis regulator [Source:HGNC Symbol;Acc:HGNC:959]" | 9323 | -0.257 | -0.2654 | No |
| 82 | IRAK1 | interleukin 1 receptor associated kinase 1 [Source:HGNC Symbol;Acc:HGNC:6112] | 9377 | -0.262 | -0.2629 | No |
| 83 | RPS6KA5 | ribosomal protein S6 kinase A5 [Source:HGNC Symbol;Acc:HGNC:10434] | 9406 | -0.265 | -0.2584 | No |
| 84 | SHC3 | SHC adaptor protein 3 [Source:HGNC Symbol;Acc:HGNC:18181] | 9608 | -0.284 | -0.2669 | No |
| 85 | MAPK10 | mitogen-activated protein kinase 10 [Source:HGNC Symbol;Acc:HGNC:6872] | 9699 | -0.291 | -0.2665 | No |
| 86 | MAP2K2 | mitogen-activated protein kinase kinase 2 [Source:HGNC Symbol;Acc:HGNC:6842] | 9932 | -0.315 | -0.2766 | No |
| 87 | MAP3K3 | mitogen-activated protein kinase kinase kinase 3 [Source:HGNC Symbol;Acc:HGNC:6855] | 10121 | -0.333 | -0.2828 | No |
| 88 | CALM1 | calmodulin 1 [Source:HGNC Symbol;Acc:HGNC:1442] | 10136 | -0.334 | -0.2754 | No |
| 89 | MAP2K1 | mitogen-activated protein kinase kinase 1 [Source:HGNC Symbol;Acc:HGNC:6840] | 10197 | -0.340 | -0.2714 | No |
| 90 | KRAS | "KRAS proto-oncogene, GTPase [Source:HGNC Symbol;Acc:HGNC:6407]" | 10544 | -0.377 | -0.2889 | Yes |
| 91 | CALM2 | calmodulin 2 [Source:HGNC Symbol;Acc:HGNC:1445] | 10579 | -0.382 | -0.2818 | Yes |
| 92 | PLCG1 | phospholipase C gamma 1 [Source:HGNC Symbol;Acc:HGNC:9065] | 10722 | -0.395 | -0.2829 | Yes |
| 93 | SH2B1 | SH2B adaptor protein 1 [Source:HGNC Symbol;Acc:HGNC:30417] | 10769 | -0.401 | -0.2762 | Yes |
| 94 | IRAK4 | interleukin 1 receptor associated kinase 4 [Source:HGNC Symbol;Acc:HGNC:17967] | 10874 | -0.413 | -0.2739 | Yes |
| 95 | MAP3K5 | mitogen-activated protein kinase kinase kinase 5 [Source:HGNC Symbol;Acc:HGNC:6857] | 10923 | -0.421 | -0.2669 | Yes |
| 96 | PIK3R2 | phosphoinositide-3-kinase regulatory subunit 2 [Source:HGNC Symbol;Acc:HGNC:8980] | 10956 | -0.425 | -0.2586 | Yes |
| 97 | PRDM4 | PR/SET domain 4 [Source:HGNC Symbol;Acc:HGNC:9348] | 11098 | -0.443 | -0.2583 | Yes |
| 98 | PIK3R1 | phosphoinositide-3-kinase regulatory subunit 1 [Source:HGNC Symbol;Acc:HGNC:8979] | 11099 | -0.443 | -0.2470 | Yes |
| 99 | GSK3B | glycogen synthase kinase 3 beta [Source:HGNC Symbol;Acc:HGNC:4617] | 11157 | -0.451 | -0.2400 | Yes |
| 100 | PTPN11 | protein tyrosine phosphatase non-receptor type 11 [Source:HGNC Symbol;Acc:HGNC:9644] | 11260 | -0.465 | -0.2361 | Yes |
| 101 | NFKBIB | NFKB inhibitor beta [Source:HGNC Symbol;Acc:HGNC:7798] | 11277 | -0.468 | -0.2254 | Yes |
| 102 | FOXO3 | forkhead box O3 [Source:HGNC Symbol;Acc:HGNC:3821] | 11305 | -0.472 | -0.2155 | Yes |
| 103 | IRS2 | insulin receptor substrate 2 [Source:HGNC Symbol;Acc:HGNC:6126] | 11390 | -0.485 | -0.2098 | Yes |
| 104 | YWHAB | tyrosine 3-monooxygenase/tryptophan 5-monooxygenase activation protein beta [Source:HGNC Symbol;Acc:HGNC:12849] | 11488 | -0.500 | -0.2046 | Yes |
| 105 | MAPK11 | mitogen-activated protein kinase 11 [Source:HGNC Symbol;Acc:HGNC:6873] | 11501 | -0.501 | -0.1928 | Yes |
| 106 | PDPK1 | 3-phosphoinositide dependent protein kinase 1 [Source:HGNC Symbol;Acc:HGNC:8816] | 11839 | -0.557 | -0.2050 | Yes |
| 107 | IRS1 | insulin receptor substrate 1 [Source:HGNC Symbol;Acc:HGNC:6125] | 11841 | -0.557 | -0.1909 | Yes |
| 108 | ATF4 | activating transcription factor 4 [Source:HGNC Symbol;Acc:HGNC:786] | 11999 | -0.593 | -0.1880 | Yes |
| 109 | SOS1 | SOS Ras/Rac guanine nucleotide exchange factor 1 [Source:HGNC Symbol;Acc:HGNC:11187] | 12041 | -0.603 | -0.1759 | Yes |
| 110 | MAPKAPK2 | MAPK activated protein kinase 2 [Source:HGNC Symbol;Acc:HGNC:6887] | 12094 | -0.619 | -0.1642 | Yes |
| 111 | YWHAE | tyrosine 3-monooxygenase/tryptophan 5-monooxygenase activation protein epsilon [Source:HGNC Symbol;Acc:HGNC:12851] | 12137 | -0.628 | -0.1515 | Yes |
| 112 | NTRK3 | neurotrophic receptor tyrosine kinase 3 [Source:HGNC Symbol;Acc:HGNC:8033] | 12157 | -0.632 | -0.1369 | Yes |
| 113 | MAP2K7 | mitogen-activated protein kinase kinase 7 [Source:HGNC Symbol;Acc:HGNC:6847] | 12209 | -0.649 | -0.1243 | Yes |
| 114 | IRAK3 | interleukin 1 receptor associated kinase 3 [Source:HGNC Symbol;Acc:HGNC:17020] | 12383 | -0.703 | -0.1200 | Yes |
| 115 | CDC42 | cell division cycle 42 [Source:HGNC Symbol;Acc:HGNC:1736] | 12681 | -0.852 | -0.1215 | Yes |
| 116 | SH2B2 | SH2B adaptor protein 2 [Source:HGNC Symbol;Acc:HGNC:17381] | 12769 | -0.930 | -0.1046 | Yes |
| 117 | JUN | "Jun proto-oncogene, AP-1 transcription factor subunit [Source:HGNC Symbol;Acc:HGNC:6204]" | 12813 | -0.994 | -0.0827 | Yes |
| 118 | NFKBIA | NFKB inhibitor alpha [Source:HGNC Symbol;Acc:HGNC:7797] | 12817 | -1.001 | -0.0574 | Yes |
| 119 | RAPGEF1 | Rap guanine nucleotide exchange factor 1 [Source:HGNC Symbol;Acc:HGNC:4568] | 12860 | -1.098 | -0.0327 | Yes |
| 120 | AKT2 | AKT serine/threonine kinase 2 [Source:HGNC Symbol;Acc:HGNC:392] | 12896 | -1.404 | 0.0003 | Yes |
Table: GSEA details [plain text format]

  

Fig 2: KEGG\_NEUROTROPHIN\_SIGNALING\_PATHWAY      
 Blue-Pink O' Gram in the Space of the Analyzed GeneSet

  

Fig 3: KEGG\_NEUROTROPHIN\_SIGNALING\_PATHWAY: Random ES distribution      
 Gene set null distribution of ES for **KEGG\_NEUROTROPHIN\_SIGNALING\_PATHWAY**
